# A superabsorbent polymer-containing wound dressing efficiently sequesters MMPs and inhibits collagenase activity in vitro

**DOI:** 10.1007/s10856-013-4990-6

**Published:** 2013-06-25

**Authors:** Cornelia Wiegand, Uta-Christina Hipler

**Affiliations:** Department of Dermatology, University Medical Center Jena, Erfurter Str. 34, 07740 Jena, Germany

## Abstract

Superabsorbent polymer (SAP)-containing wound dressings present a valuable and unique category of wound management products. An in vitro approach was used to assess the effects of a new SAP dressing in treatment of non-healing wounds. It was shown that the SAP dressing possesses a significant binding capacity for MMP-2 and MMP-9 in vitro (*P* < 0.001). The inclusion of the bound proteases was so strong that no MMP-2 and only marginal amounts of MMP-9 were released from the dressing samples in a subsequent elution step. In addition, the SAP dressing was able to take up collagenase and reduce its activity in vitro. However, collagenase was not completely inactivated upon binding and enzyme-mediated substrate turnover could be observed at the dressings. In conclusion, in vitro data confirm the positive effect of the SAP wound dressing observed in vivo. The findings suggest that it should be specifically useful for highly exuding wounds with an elevated proteolytic activity that needs to be reduced to support healing.

## Introduction

Non-healing wounds, like venous, pressure and diabetic ulcer, have become a rising charge to society as an increasing number of patients suffer from wounds that fail to heal. Several studies have shown that exudates from these chronic wounds are characterized by elevated levels of matrix metalloproteinases (MMPs) [1, 2], contain excessive amounts of polymorphonuclear granulocyte-derived elastase (PMN elastase) [3–5], and high concentrations of free radicals [6]. These inflammatory mediators shift the balance of matrix synthesis and its degradation towards tissue destruction. The disproportionate action of proteases leads to considerable reduced amounts of growth factors [7] and proteinase inhibitors like tissue inhibitors of matrix metalloproteases [8]. Hence, modern wound management focuses on reduction of these inflammatory mediators and creation of a moist wound environment. Both should aid in establishing a physiological wound milieu and promote wound healing. For this purpose, a diversity of occlusive dressings is available in various forms, such as films, foams, or gels, made from different materials like alginates, polyurethane, hyaluronic acid, and collagen. However, highly exuding wounds may require other options as not all of these materials are able to handle an excess amount of exudate whose control is critical in the management of chronic wounds [9]. Therefore, wound dressings containing superabsorbent polymers (SAP) were devised. SAPs are able to take up a multiple amount of water of their own dry weight and are mainly utilized as an absorbent for water and aqueous solutions in diapers, adult incontinence products, feminine hygiene products, and similar applications [10]. They are mainly fabricated from acrylic acid and a crosslinker by solution or suspension polymerization [10]. The resulting polyacrylate superabsorbers have a high density of ionic charges which accounts for the hydroactive properties [10, 11] and the protein binding capacity [12–14]. Polyacrylate containing wound dressings have been shown to be particularly effective in the cleansing phase where the proteolytic activity is high and leading to tissue breakdown [13–15]. A recent study by Eming et al. [13] could show that polyacrylate superabsorbers can inhibit MMP activity in vitro and ex vivo. Moreover, it was found that SAP dressings exhibit a high binding capacity for PMN elastase and are able to inhibit free radical formation in vitro [12].

**Fig. 1:**
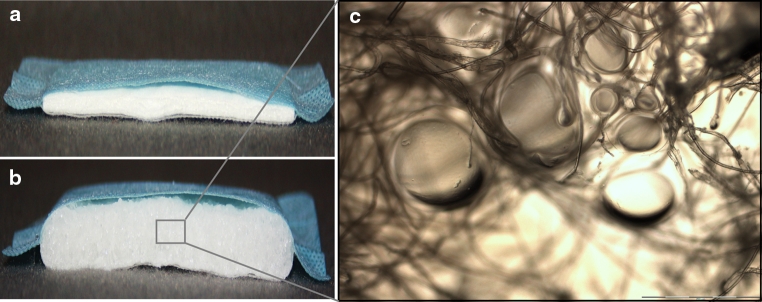
The SAP-containing wound dressing consists of a 4-layer construction (**a**): wound contact layer, distribution layer, absorbent core, and a fluid repellent cover sheet (*blue*). Upon contact with fluid the absorbent core begins to swell (**b**). It is comprised of a cellulose fiber matrix enclosing the SAP particles (**c**, light microscopic image, ×20 magnification)

The aim of this study was to investigate the binding properties of a new SAP-containing wound dressing (Fig. 1) for MMPs. Moreover, the capacity of the SAP wound dressing to inhibit collagenase activity was analyzed in vitro.

## Materials and methods

### Materials

The tested SAP-containing dressing Mextra™ Superabsorbent was obtained from the manufacturer, Molnlycke Healthcare Group (UK). Human MMP-2 and MMP-9 were taken from the specific enzyme immunoassays (ELISA) from R&D Systems GmbH (Germany). Collagenase was purchased from Invitrogen (Germany).

### Binding of MMP-2 and MMP-9

Lyophilized MMP-2 and MMP-9 (R&D systems GmbH, Germany) were reconstituted as recommended in the manufacturer’s instructions. For further experiments reconstituted MMP-2 was diluted to a concentration of 5,000 pg/mL while MMP-9 was diluted to a concentration of 2,000 pg/mL. Wound dressing samples were cut using 8 mm punch biopsies (Stiefel Laboratorium GmbH, Germany) corresponding to 0.5 cm^2^. The samples were placed into 24-well cell culture plates (Greiner bio-one, Germany). Glass cover slips (0.5 cm^2^) were used as controls. Each specimen was taken in a final volume of 1 mL of protease solution. Samples were incubated up to 24 h at 37 °C on a plate mixer (THERMOstar, BMG Labtech GmbH, Germany). After incubation, supernatants were collected, immediately frozen and stored at −20 °C until testing. Subsequently, bound protein was eluted from the individual wound dressing samples by shaking in 1 mL PBS for 1 h at 37 °C. For determination of the MMP-2 and the MMP-9 concentrations, the specific ELISAs were purchased from R&D Systems GmbH (Germany) and run as recommended by the manufacturer. Optical density was measured at 450 nm with a reference measurement at 620 nm using a plate photometer (BMG Labtech GmbH, Germany). Subsequently, the protease concentrations were evaluated according to a 4-parameter-fit with lin–log coordinates for optical density (linear scale) and concentration (logarithmic scale).

### Inhibition of collagenase activity

Lyophilized collagenase was reconstituted as recommended in the manufacturer’s instructions (EnzChek Collagenase/Gelatinase assay kit, Invitrogen, Germany). A 1,000 U/mL stock solution of collagenase was prepared in distilled water. For experiments, the stock solution was diluted to 0.2 U/mL in the reaction buffer (0.1 M Tris–HCl, pH 8.0, containing 0.2 mM sodium azide and 0.5 % bovine serum albumin). Wound dressing samples were cut using 8 mm punch biopsies (Stiefel Laboratorium GmbH, Germany) corresponding to 0.5 cm^2^. The samples were placed into 24-well cell culture plates (Greiner bio-one, Germany). Glass cover slips (0.5 cm^2^) were used as controls. Each specimen was taken in a final volume of 1 mL of protease solution. Samples were incubated up to 24 h at 37 °C on a plate mixer (THERMOstar, BMG Labtech GmbH, Germany). After incubation, supernatants were collected, immediately frozen and stored at −20 °C until testing. Subsequently, bond protein was eluted from the individual wound dressing samples by shaking in 1 ml PBS for 1 h at 37 °C. Collagenase activity was determined using the Collagenase/Gelatinase assay kit (Invitrogen, Germany). The assay was run as recommended in the instructions. Briefly, 80 μL reaction buffer and 20 μL DQ gelatin substrate were added, followed by the injection of 100 μL sample. The fluorescence was measured continuously for 1 h at room temperature using a fluorescence plate reader (FLUOstar Galaxy, BMG Labtech; excitation wavelength: 495 nm, emission wavelength: 538 nm). Additionally, eluted dressing samples were incubated for 1 h with 800 μL reaction buffer and 200 μL DQ gelatin. Afterwards, 200 μL samples were transferred to a black 96-well plate and substrate turnover was measured by fluorescence intensity (excitation wavelength: 495 nm, emission wavelength: 538 nm) using a fluorescence plate reader (FLUOstar Galaxy, BMG Labtech).

### Statistical analysis

All values cited are expressed as means ± SE (standard error). One-way analysis of variance was carried out to determine statistical significances (Microsoft^®^ Excel 2000). Differences were considered statistically significant at a level of *P* < 0.05. Asterisks indicate significant deviations from the control at the respective incubation time (*P < 0.05; **P < 0.01; ***P < 0.001).

## Results

**Fig. 2:**
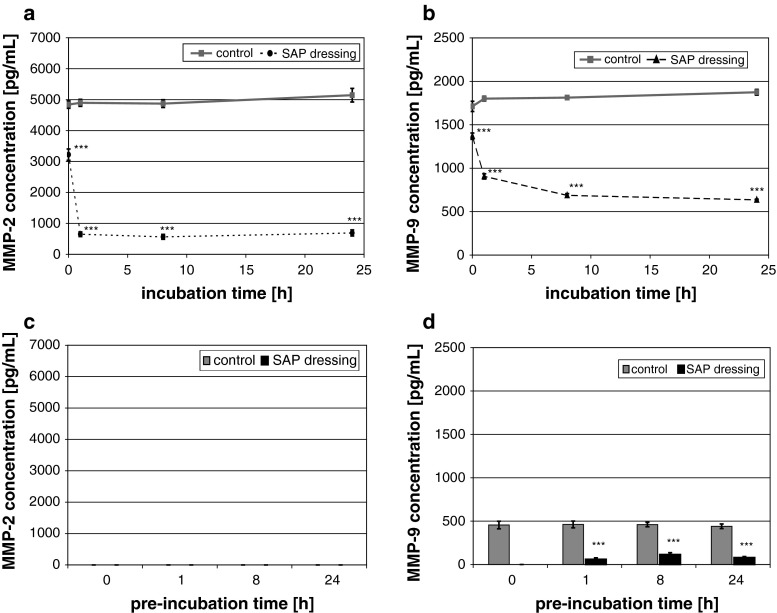
The SAP-containing wound dressing reduces the MMP-2 (**a**) and MMP-9 (**b**) concentration in a defined enzyme solution. No MMP-2 was detected in the eluate (**c**). Only marginal amounts of MMP-9 could be eluted from the samples (**d**)

**Fig. 3:**
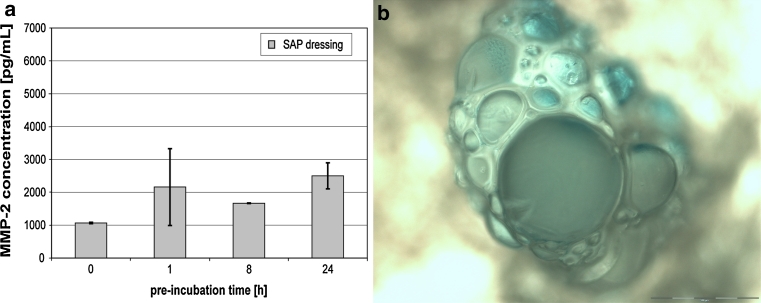
MMP-2 could be detected in the disrupted dressing samples by direct incubation with the respective ELISA antibodies (**a**). Light microscopy of the samples (×20 magnification) revealed that MMP-2 is bound to the cortical zone of the particles, distinguishable by the *light blue color* development (**b**)

**Fig. 4:**
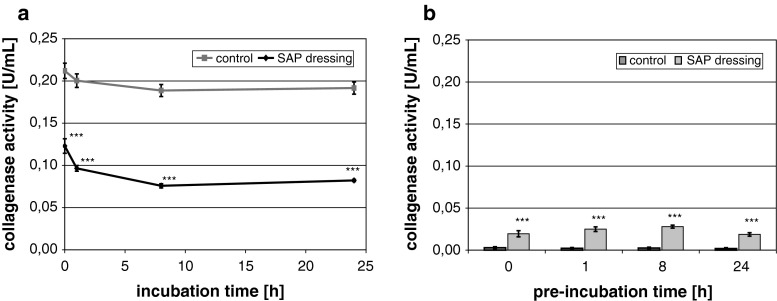
Collagenase activity was significantly reduced by the SAP dressing (**a**). No collagenase activity was observed in the eluate. However, collagenase was not inactivated by binding to the SAP; a significant substrate turnover could be noted when the dressings were incubated with the collagenase substrate DQ gelatin (**b**)

**Fig. 5:**
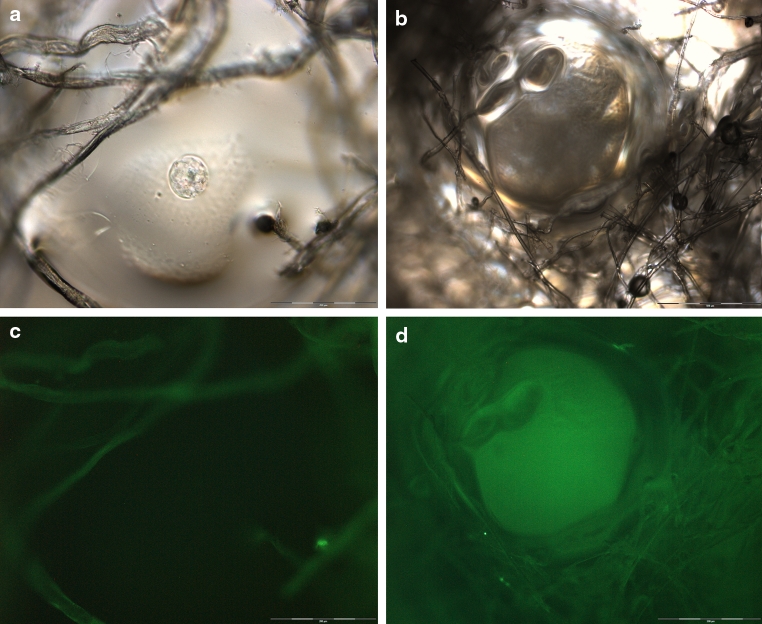
Light microscopic images of the SAP dressing show the fibers and fluid swollen polyacrylate particles of the absorbent layer (**a**, **b**). The fibers exhibit a slight intrinsic fluorescence but the polyacrylate particle is not visible in fluorescence imaging (ex: 485 nm, em: 520 nm) (**c**). Polyacrylate particles with bound collagenase became visible in fluorescence imaging (ex: 485 nm, em: 520 nm) after incubation with collagenase substrate (**d**). Hence, it can be concluded that the SAP beads in the dressing bind the active enzyme and efficiently remove it from the solution, but collagenase remained at least partially active. (×20 magnification)

The SAP dressing tested exhibited a significant binding capacity for MMP-2 in vitro (*P* < 0.001; Fig. 2a). The dressing samples quickly reduced the amount of MMP-2 in the supernatant upon contact. Moreover, no protein was detected in the eluate (Fig. 2c). It was observed that the protease was so tightly bound by the SAP dressing samples that even disruption of the dressing and aggressive elution techniques (e.g. vortexing or ultrasonic bath) did not lead to the release of MMP-2 in vitro. However, bound MMP-2 could be perceived by direct incubation of the disrupted dressing samples with the respective ELISA antibodies (Fig. 3a). Moreover, these samples were subjected to light microscopy, where binding of MMP-2 was verified by the blue color of the TMB-substrate (Fig. 3b). It could be shown that MMP-2 is bound to the cortical zone of the polyacrylate particles. In addition, the SAP dressing was found to significantly bind MMP-9 in vitro (*P* < 0.001; Fig. 2b). Only a marginal amount of the protease was released from the dressings in the subsequent elution step compared to the control (Fig. 2d). Moreover, a significant effect of the SAP dressing on the activity of collagenase in the supernatant was observed (*P* < 0.001; Fig. 4a). Almost no enzyme activity was detectable in the eluate (data not shown). However, it could be demonstrated that the collagenase bound to the dressing samples remains at least partially active (Fig. 4b). An enzyme-mediated substrate turnover could be observed directly at the SAP beads of the dressing (Fig. 5).

## Discussion

Exudate control is crucial in management of chronic wounds [9]. The excessive fluid needs to be removed from the wound (liquid acquisition), quickly dispersed in the dressing (liquid distribution), and maintained inside the material (liquid retention). The highest liquid retention can be achieved with cross-linked, high molecular-weight polyelectrolytes, called SAPs [11]. Most SAPs are cross-linked networks of flexible polymer chains with abundant ionizable carboxyl groups, typically joined with sodium ions [10, 11]. Fluid absorption occurs through osmotic pressure caused by the concentration gradient of electrolytes inside and outside the SAP particles [11]. This process is accompanied by the detachment of the sodium ion, which leaves the charged carboxyl group behind [10], and the unfolding of the macromolecule chains, due to the repulsion forces of the negatively charged sites [11]. This allows the SAP to swell and absorb large amounts of liquid. During this process, SAPs also take up and retain proteins, cell debris and even micro-organisms [12–15]. Binding of proteins is most likely due to electrostatic forces that arise between their positively charged groups and the negatively charged carboxyl groups of the polyacrylate. It seems likely that the SAP dressing tested binds and removes MMPs from the test solution by the same mechanism, e.g. Eming et al. [13] observed in their experiments a significant reduction of MMP concentration. Moreover, SAPs have been shown to reduce the concentration of PMN elastase by electrostatic interactions [12]. Likewise, MMPs are known to be bound to other polymers, such as oxidized regenerated cellulose, collagen or alginate, by electrostatic forces [16–18]. In accordance, it was found that the SAP dressing tested exhibited a significant binding capacity for MMP-2 and MMP-9 in vitro. Most remarkably was the fast kinetic of the attachment of the protease to the SAP, upon contact the enzyme concentration in solution was decreased. Moreover, binding of MMP-2 was found to be irreversible and only marginal amounts of MMP-9 could subsequently be eluted from the material. These results are similar to the outcome of a recently published study that demonstrated the binding of MMP-2 as well as MMP-9 and the inactivation of collagenase by a superabsorbant dressing [19]. Here, it could further be demonstrated that the proteases are bound to the cortical zone of the SAP particles in the absorbent layer of the dressing. These findings are in line with previous reports by Eming et al. [13]. Additional experiments revealed that incubation with the SAP dressing reduces collagenase activity by sequestration and removal of the protease from the fluid in vitro. However, collagenase activity was not inhibited as the protease remained at least partially active when bound to the dressing’s core. Comparable results were observed for binding of elastase by other SAP-containing dressings that were able to take up elastase and effectively prevent its subsequent release but were not capable to impede enzyme-mediated substrate turnover [12]. In contrast, Eming et al. [13] observed in their experiments also an indirect effect of SAP on the MMP activity by binding of essential Ca^2+^ and Zn^2+^ ions.

## Conclusion

It can be expected that the SAP dressing achieves a beneficial effect on wound healing not only by absorbing exudate but also by reducing the concentration of proteases such as MMP-2 and MMP-9. Hence, it should be particularly effective in the cleansing phase during treatment of chronic wounds where the proteolytic activity is high and tissue destruction occurs.

## Abbreviations

ECM: Extracellular matrix
MMP: Matrix metalloproteinase
PMN: Polymorphonuclear
SAP: Superabsorbent polymer
TIMP: Tissue inhibitor of matrix metalloproteases
